# Plant Abandonment by *Busseola fusca* (Lepidoptera: Noctuidae) Larvae: Do Bt Toxins Have an Effect?

**DOI:** 10.3390/insects11020077

**Published:** 2020-01-22

**Authors:** Andri Visser, Hannalene Du Plessis, Annemie Erasmus, Johnnie Van den Berg

**Affiliations:** 1 Unit for Environmental Sciences and Management, IPM program, North-West University, Potchefstroom 2520, South Africa; visseran3@gmail.com (A.V.); hannalene.duplessis@nwu.ac.za (H.D.P.); 2 Agricultural Research Council, Grain Crops, Private Bag X1251, Potchefstroom 2520, South Africa; ErasmusA@arc.agric.za

**Keywords:** *Busseola fusca*, larval migration, Bt maize, insect resistance management, insect behavior

## Abstract

*Busseola fusca* (Fuller; Lepidoptera: Noctuidae) is an important pest of maize in Africa and can be effectively controlled by Bt maize. However, the sustainability of this technology is threatened by resistance evolution, which necessitates the implementation of the high-dose/refuge insect resistance management (IRM) strategy. Despite the success of this IRM strategy, it is based on several assumptions about insect-hostplant interactions that are not always valid for different pest species. In this study, the plant abandonment behavior of Cry1Ab-resistant and susceptible *B. fusca* larvae were evaluated on a non-Bt, single toxin (*Cry1Ab*), and a pyramid event (*Cry1.105* + *Cry2Ab2*) of maize over a four-day period. The aim was to determine if larvae are more likely to abandon maize plants that contain Bt-toxins than conventional non-Bt plants, and if resistance to the Cry1Ab-toxin affects this behavior. This study found that both Bt-resistant and susceptible *B. fusca* neonate larvae show feeding avoidance behavior and increased plant abandonment rates when exposed to Bt maize leaf tissue. The implications of these findings for the design of IRM strategies and choice of refuge structures are discussed in the context of Bt maize in Africa.

## 1. Introduction

Since the commercial release of genetically engineered Bt maize in South Africa in 1998 [1,2], this technology has been used as an effective control method against *Busseola fusca* (Fuller; Lepidoptera: Noctuidae) [3], a major pest of maize and sorghum in Africa [4,5,6].

The sustainability of Bt maize technology is threatened by the evolution of resistance by pest populations [7,8,9,10,11] due to the sustained selection pressure exerted throughout the growing season [11,12,13]. A mere eight years after the release of Bt maize in South Africa, *B. fusca* populations started to exhibit field-evolved resistance to Cry 1Ab maize [14]. However, it is possible to deploy Bt crops sustainably, if an effective insect resistance management (IRM) strategy is in place [13,15]. 

The high-dose/refuge (HDR) approach is one of the most widely applied IRM strategies [8,12,16]. This strategy calls for the combined use of Bt toxins with a level of expression that kills heterozygous-resistant individuals, and a source of non-Bt host plants (refuge area) near the Bt field [17,18]. This refuge acts as a source of homozygous susceptible individuals. Consequently, the rare homozygous resistant individuals that survive on the Bt crop mate with the abundant homozygous susceptible individuals that are produced by plants in the refuge area. The proportion of resistance genes that are present in the subsequent generation is therefore limited, since the heterozygous offspring of these mating pairs are eliminated by the high-dose of Bt toxins [13,18,19,20,21]. 

A non-Bt refuge area can be planted as a separate block, strips within the Bt field, or as a perimeter surrounding the Bt field (structured refuge) [13]. Unstructured refuges, or seed mixtures, refer to the planting of a mixture (with a predetermined ratio) of non-Bt and Bt seed [22,23,24,25]. The implementation of structured refuges is complicated in small farming environments [26,27]. The use of unstructured refugia and wild host plants as refuges in an IRM strategy for African stem borers, is often suggested as a possible solution [28,29,30]. Van den Berg [26] did however indicate that reliance on wild host plants as refuge in most of the developing world is not appropriate to small farming systems and, in the case of B. fusca, that these wild host plants are very limited and do not sustain large enough numbers of this pest.

To ensure the optimal functioning of the HDR-strategy, the refuge structure must be suited to the biology and behavior of the specific target pest [31,32,33]. Should the larval stage of the target pest be highly mobile, the use of a seed mixture approach may result in rapid evolution of resistance, since this would lead to larvae being exposed to sub-lethal doses of Bt toxins [23,34,35,36]. Brévault et al. [37] reported that another pest species such as *Helicoverpa zea* (Lepidoptera: Noctuidae), which is inherently tolerant to Bt toxins and has mobile larvae, might evolve resistance to Bt cotton more rapidly in fields planted with seed mixtures. The latter authors reported that resistance might evolve two to 4.5-fold faster in the seed mixture relative to separate blocks of Bt and non-Bt cotton. 

The behavioral traits of *B. fusca* that could impact resistance evolution are the preference of gravid female moths for ovipositional hosts and larval feeding preference for the host plant (including the ensuing larval migration behavior) [21,38]. Preferences are based on the chemical (e.g., nutrient content) and physical (e.g., trichomes) host plant characteristics [39,40]. It is therefore possible that the presence of Bt-toxins in maize leaf tissue can be detected by foraging larvae and that this may affect their feeding and migration behavior. When a host plant is not preferred by larvae, foraging and movement within and between plants is likely to continue [41,42]. Refuge design should therefore aim to mitigate the selection pressure on migrating larvae. 

Larval migration generally takes two forms: crawling or ballooning [39]. Ballooning, also referred to as silking, is when larvae produce silk strings to dangle themselves from plant structures until they come into contact with a different plant/plant structure or are carried off by the wind [23,32]. As larvae mature, ballooning becomes increasingly difficult due to their size and weight. Larger larvae therefore disperse mostly by crawling [36]. Larval density, host plant suitability and environmental conditions are all factors that could influence the rate and success of larval migration [32,42].

It is essential that IRM approaches are tailored to best fit the behavior of the target pest species and the scale of production [8,43], especially in smallholder and subsistence agricultural systems, which are predominant in developing regions such as Africa [6,44,45]. A generic IRM strategy developed for use in industrial agricultural systems will most likely not be effective in these developing regions, due to the challenges provided by implementing structured refuges [45,46,47,48,49]. Therefore, information about the target pest migration behavior is crucial to develop practical and appropriate IRM strategies.

Visser et al. [41] investigated the effect that resistance to Cry1Ab maize has on the oviposition and feeding preference of *B. fusca* moths and larvae. Although no differential oviposition preference was observed for either Bt-resistant or susceptible female moths, neonate larvae were able to detect Bt toxins and displayed feeding avoidance behavior on Bt maize leaf samples. This paper aims to expand the results of Visser et al. [41] by determining if larvae are more likely to abandon maize plants that contain Bt-toxins than conventional non-Bt plants, and if resistance to the Cry1Ab-toxin affects this behavior. 

## 2. Materials and Methods

### 2.1. Stock Colonies of B. fusca

A Cry1Ab-susceptible population (EC18-S) collected in the Eastern Cape region as well as a Cry1Ab-resistant population collected in the Harrismith region (HAR18-R) were used in this study. The Cry1Ab-susceptible population (EC18-S) of diapausing *B. fusca* larvae were collected from maize stubble in a non-Bt field (S 33°4′28.153″; E 27°38′41.204″) during late August 2018 in the Eastern Cape province, where *B. fusca* larvae are reported to be highly susceptible to Cry1Ab maize [50,51]. The larvae were placed in 15 L plastic containers (37 cm (L) × 29 cm (W) × 19 cm (H)), with 50 larvae per container. The containers were kept in a temperature-controlled room at 24 ± 2 °C, ambient humidity and a 14L:10D photoperiod. Sheets of paper were placed inside the containers to provide shelter for the larvae. The contents of each container were sprayed generously with distilled water daily to terminate diapause and initiate pupation, following the methods described by Van Rensburg and Van Rensburg [52].

Larvae of the HAR18-R population were collected from a maize field (growth stages V6–V10) in the Harrismith area (S 28°12′54″; E 29°4′51.3″) during January 2018. These larvae were kept in similar containers and environmental conditions as described above. Larvae were reared to the pupal stage on non-Bt maize stems. 

The status of resistance/susceptibility of each population was confirmed prior to the study, by means of a bioassay comparing larval survival on MON810 and non-Bt maize. A total of 20 neonate larvae were taken from the egg batches of ten different female moths of each population. These larvae were then divided into two plastic containers (100 mL, 10 larvae in each container) and provided with whorl leaf material from either non-Bt or MON810 maize plants (4-week-old plants). Thus, a total of 100 neonate larvae were placed on non-Bt material, and 100 larvae were placed on MON810 material. The containers were kept under the same conditions as described above. After 10 days, the number of surviving larvae on both non-Bt and MON810 maize were determined. The resistant population had a survival percentage of 89% on MON810 and 88% on non-Bt, whereas only 8% of the susceptible population survived on MON810 maize (with the survivors showing reduced growth), compared to 91% survival on non-Bt maize.

### 2.2. Production of Neonate B. fusca Larvae for Experiments

*Busseola fusca* egg batches were obtained by rearing the field-collected larvae according to the methods described in Visser et al. [41]. The egg batches were placed in 50 mL plastic containers with mesh-infused lids, which were then kept in a glass desiccator (30 cm diameter) with the RH maintained at 70% ± 5% by means of a potassium hydroxide solution [53]. The desiccator was kept in a rearing chamber (26 °C ± 1 °C, RH 65% ± 5%, and a photoperiod of 14L:10D) and was checked daily for hatching neonate larvae. These first-generation neonate larvae were used in the experiments. 

### 2.3. Maize Hybrids

Three maize hybrids representing two different Bt treatments and a control were used in this study. These were a single-gene Bt hybrid expressing Cry1Ab protein (DKC 8012B; MON810), a pyramid hybrid expressing Cry1.105 + Cry2Ab2 proteins (DKC 8012 B GEN; MON89034), and a near-isogenic non-Bt hybrid (DKC 8010; non-Bt).

### 2.4. Experiment Protocol

Twelve plants of each of the three hybrids were used for both the Cry1Ab-resistant HAR18-R and the susceptible EC18-S populations. Each plant served as a replicate and the trial design was a randomized complete block. 

All maize plants were grown individually in 5 L plastic pots (22.5 cm × 18 cm) in a horticultural tunnel at the North-West University in Potchefstroom, South Africa. Plants were grown at ambient temperatures between October and November 2018. Plants were watered as needed and received a single application of a nutrient solution (Nutrifeed^®^, Starke Ayres (Pty) Ltd., Bloemfontein, South Africa).

This experiment was conducted in a laboratory at 26 ± 1 °C, and photoperiod of 14L:10D. Plants were in the V4-stage of development (2–3 weeks after seedling emergence, approximately 50 cm in height) when the study commenced. Each potted maize plant was inoculated with 30 neonate larvae of either the Cry1Ab-resistant or susceptible population. Thirty neonates were used per plant since this number is very close to the average egg batch size of between 28 and 33 eggs, laid by *B. fusca* females under field conditions [6]. Inoculation was done by placing the neonate larvae directly into the whorl of the maize plant by means of a fine camel hair brush. Two wooden hardboards (40 cm × 80 cm each), covered with yellow sticky trap roll (Insect Science (Pty), South Africa) were placed at the base of each plant to form an 80 cm × 80 cm catchment area to trap ballooning larvae beneath the maize plants. A small indent was cut into the lengthwise margins of the boards to accommodate the maize stem, thereby ensuring a solid catchment area with no gaps. Some of the leaves of the plants were trimmed to prevent leaf surfaces from extending past the sticky surface area. The number of larvae that abandoned plants by means of ballooning onto the sticky trap surface was recorded daily for four days.

### 2.5. Data Analysis

Data analysis was conducted using Statistica v13.3 (TIBCO Software Inc. 2017, Palo Alto, CA, USA). The number of larvae that abandoned the plants per day was analyzed using repeated measures analysis of variance (ANOVA). The data of the Cry1Ab-resistant and susceptible populations were analyzed separately (since the experiment was conducted at two different times for the populations—HAR18-R population was evaluated during March 2018 and EC18-S population during November 2018). The analyses included both the main factors of day and hybrid. A Tukey HSD post-hoc test was used to identify significant differences between means.

## 3. Results

The repeated measures ANOVA of plant abandonment data indicated that the main factors (hybrid and day) were significant (*p* < 0.05) for both the Cry1Ab-resistant and susceptible populations (Table 1), whereas the interaction between these two factors were only significant for the resistant population. The two populations were analyzed separately since the experiments with the resistant population was conducted in March 2018, whereas the susceptible population was evaluated in November 2018. 

Out of the total of 360 larvae inoculated onto plants of each of the three treatments, 19.4%, 39.4%, and 70.5% abandoned the host plants of the non-Bt, MON810, and MON89034 treatments, respectively. The number of Cry1Ab-resistant larvae that attempted to migrate away from MON89034 (M = 5.29, SE = 0.42) was significantly higher (*p* < 0.001) than that for MON810 (M = 2.96, SE = 0.42) and non-Bt (M = 1.46, SE = 0.42). The difference in mean number of migrating larvae was also significant between MON810 and non-Bt (*p* = 0.04). The plant abandonment results for the Cry1Ab-susceptible larvae on the three different hybrids followed a similar trend to that of the resistant population, where the migration from MON89034 (M = 1.6, SE = 0.28) was significantly (*p* = 0.006) higher than from non-Bt plants (M = 0.35, SE = 0.28). However, the mean number of susceptible larvae to abandon MON810 plants (M = 1.00, SE = 0.28) did not differ significantly from either that of MON89034 (*p* = 0.22) or non-Bt (*p* = 0.24). 

**Figure 1:**
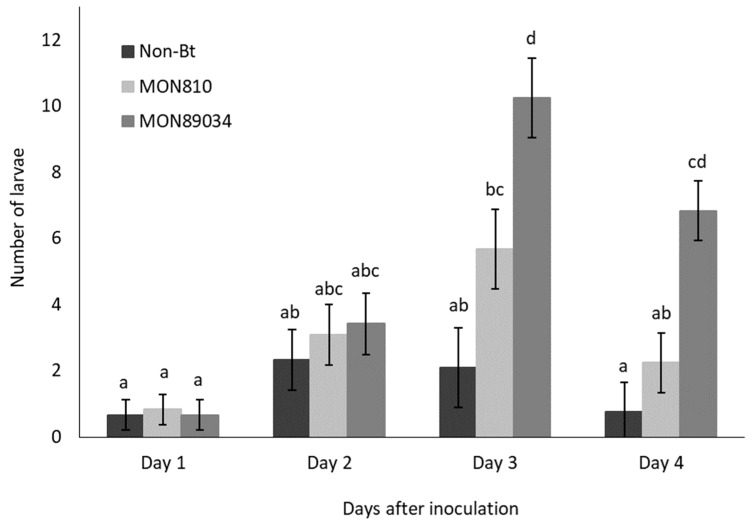
Mean (±SE) number of Bt-resistant *B. fusca* larvae to abandon maize plants per day (V4 growth-stage) over a 4-day period after inoculation onto non-Bt, MON810, and MON89034 plants. Means marked with different letters for each day-interval are significantly different (*p* < 0.05). The experiment was conducted in March 2018 and was replicated 12 times for each population, with *n* = 30 larvae inoculated per plant.

**Figure 2:**
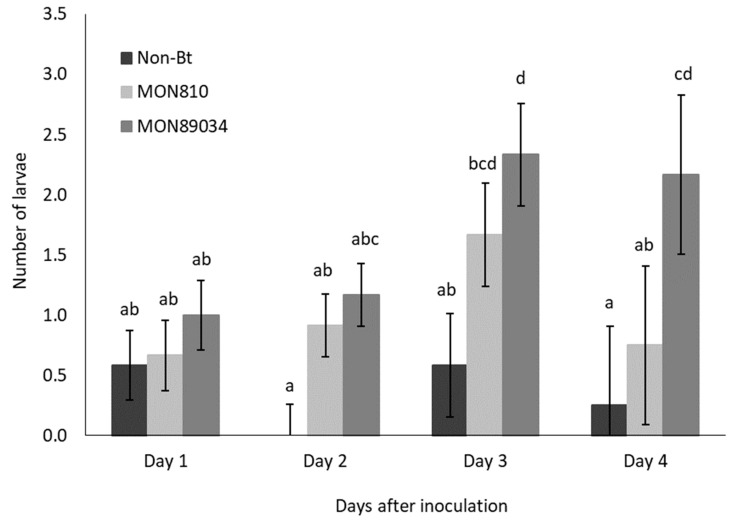
Mean (±SE) number of Bt-susceptible *B. fusca* larvae to abandon maize plants per day (V4 growth-stage) over a 4-day period after inoculation onto non-Bt, MON810, and MON89034 plants. Means marked with different letters for each day-interval are significantly different (*p* < 0.05). The experiment was conducted in November 2018 and replicated 12 times for each population, with *n* = 30 larvae inoculated per plant.

The rate of ballooning off plants was higher for Cry1Ab-resistant larvae than susceptible larvae, but the migration of both populations followed the same trend and peaked by Day 3 and Day 4 after inoculation (Figure 1 and Figure 2). The rate at which the Cry1Ab-resistant larvae ballooned off the MON89034 plants increased over time and was consistently higher compared to the other maize hybrids by Day 3 and Day 4 after inoculation (Figure 1). 

## 4. Discussion

Larval ballooning off plants occurred irrespective of whether plants had the Bt trait or not. This can be attributed to a larval pre-feeding movement phase described by Zalucki et al. [42], in their review article on the ecology and behavior of first instar Lepidoptera larvae. Zalucki et al. [42] reported that some larvae that hatch from an egg mass undertook long distance dispersal via ballooning, often before feeding on the natal host plant. This behavior has been observed in several lepidopteran families (including Noctuidae, such as *B. fusca*) and is thought to be largely genetically determined [23,54,55,56]. Thus, a proportion of larvae will always abandon the natal host plant, regardless of host quality. However, poor host quality may often lead to an increase in the number of larvae that abandon the natal plant [42,57].

The number of *B. fusca* larvae abandoning the inoculated plant, and the rate of their migration, was higher on both the Bt hybrids when compared to the non-Bt hybrid. This is most likely due to Bt toxin avoidance behavior displayed by lepidopteran larvae [58]. Visser et al. [41] also reported that both the Cry1Ab-resistant and susceptible larvae displayed increased toxin avoidance behavior to MON810 plants, but still reacted markedly more to MON89034 than MON810 plants. Razze and Mason [23] conducted a similar study in which they monitored the migration behavior of *Ostrinia nubilalis* (Hübner; Lepidoptera: Crambidae) larvae on maize plants under both field and laboratory conditions. They evaluated neonate larval dispersal from non-Bt sweetcorn, two single-gene Bt maize hybrids (*Cry1F* and *Cry1Ab*) and a pyramid maize cultivar (*Cry1F*, *Cry1Ab*, and *Cry34*/*35Ab1*) for four hours, and found that larval dispersal rate was significantly higher on Bt maize treatments compared to the non-Bt sweetcorn.

The Bt avoidance behavior exhibited by larvae is virtually ubiquitous in lepidopteran pest species [58]. Visser et al. [41] summarized the studies that investigated this phenomenon in Lepidoptera pest species and reported that the neonate larvae of 16 species displayed significant Bt-toxin avoidance behavior. There are however reports of Lepidoptera species that remain on Bt host plants even though the larvae are susceptible to Bt toxins. For example, larvae of *Spodoptera frugiperda* (J.E. Smith; Noctuidae) [57], *Helicoverpa armigera* (Hübner; Noctuidae) [59], *Plutella xylostella* (L.; Plutellidae) [60,61,62], and *Hyphantria cunea* (Drury; Erebidae) [63] have been reported not to display Bt toxin avoidance behavior. Similar to Visser et al. [41], the Cry1Ab-resistant population displayed a greater proclivity for migration. This could be due to any one of several factors such as possible secondary effects of the resistance trait, or the parental generation (the susceptible EC18-S population were derived from diapause larvae, whereas the resistant HAR18-R population originated from a late-season generation) [41]. Berdegué et al. [64] provided another explanation for this phenomenon, i.e., susceptible larvae become immobile soon after ingesting the Bt toxins expressed in the maize plants and are therefore unable to migrate to neighboring plants. However, this phenomenon does not explain why Cry1Ab-resistant larvae also dispersed at a higher rate from non-Bt maize plants than Cry1Ab-susceptible larvae, which merits further investigation in future studies.

It is unlikely that the differential reaction of Bt-resistant and susceptible larvae to the presence of Bt toxins in plant tissue can be ascribed to genetic differences that existed between the geographically different populations that were used in the study. *Busseola fusca* has a very limited host plant range and in South Africa basically only feeds on maize, which limits the possibility of development of host-associated strains of this pest [26]. Furthermore, *B. fusca* populations in maize in different regions of South Africa are in contact with each other and continued gene-flow occurs between them [65]. Molecular markers reflected extensive gene flow among populations indicating a largely homogenous population [66] in which haplotypes are not restricted to particular geographic regions, but instead have a wide geographic distribution [67].

## 5. Conclusions

This study improves our understanding of *B. fusca* larval host preference and migration behavior on maize and will contribute valuable information to aid the design of resistance management strategies for this pest in Africa.

The results of this study support the observation made by Visser et al. [41] and found that both resistant and susceptible *B. fusca* neonate larvae show feeding avoidance behavior and increased plant abandonment when exposed to Bt maize leaf tissue. Since seed mixtures are a suboptimal IRM strategy for pests that are highly mobile during the larval stage, these findings suggest that structured refuges should be used when the target pest of Bt maize is *B. fusca*. Therefore, future studies should aim to establish whether the increase in plant abandonment due to toxin avoidance behavior would lead to greater larval migration within maize fields, which could impact the viable IRM strategies available to both commercial and smallholder maize farmers in Africa. 

## Figures and Tables

**Table 1:** Repeated measures analysis of variance of the plant abandonment test conducted with neonate *B. fusca* larvae of a Bt-resistant (conducted in March 2018) and susceptible (conducted in November 2018) population. The number of larvae that abandoned maize plants of three treatments was recorded daily for 4 days after inoculation. The treatments were non-Bt (DKC 8010), MON810 (DKC 8012 B), and MON89034 (DKC 8012 B GEN).

	Source	SS	d.f.	MS	F-Value	*p*-Value
**Susceptible population**	Hybrid	41.35	2	20.67	5.57	**
	Day	15.74	3	5.25	2.85	*
	Hybrid * Day	11.32	6	1.89	1.03	NS
	Error	182.19	99	1.84		
**Resistant population**	Hybrid	358.22	2	179.11	21.47	***
	Day	505.634	3	168.55	16.04	***
	Hybrid * Day	292.61	6	48.77	4.64	***
	Error	1040.25	99	10.51		

Significance indicated by NS (not significant), * (*p* < 0.05), ** (*p* < 0.01), and *** (*p* < 0.001).

## References

[1] Van den Berg J., Van Rensburg J.B.J. (1996). Effect of various directional insecticide sprays against *Busseola fusca* (Lepidoptera: Noctuidae) and *Chilo partellus* (Lepidoptera: Pyralidae) in maize and sorghum. S. Afr. J. Plant Soil..

[2] Van den Berg J., Nur A.F., Polaszek A. (1998). Chemical control. Cereal Stem Borers in Africa: Economic Importance, Taxonomy, Natural Enemies and Control.

[3] Van den Berg J., Van Wyk A. (2007). The effect of Bt maize on *Sesamia calamistis* in South Africa. Entomol. Exp. Appl..

[4] Kfir R., Overholt W.A., Khan Z.R., Polaszek A. (2002). Biology and management of economically important lepidopteran cereal stem borers in Africa. Annu. Rev. Entomol..

[5] Tounou A.K., Gounou S., Borgemeister C., Goumedzoe Y.M.D., Schulthess F. (2010). Susceptibility of *Eldana saccharina* (Lepidoptera: Pyralidae), *Busseola fusca* and *Sesamia calamistis* (Lepidoptera: Noctuidae) to *Bacillus thuringiensis* Cry toxins and potential side effects on the larval parasitoid *Cotesia sesamiae* (Hymenoptera: Braconidae). Biocontrol. Sci. Technol..

[6] Calatayud P.A., Le Rü B., Van den Berg J., Schulthess F. (2014). Ecology of the African maize stalk borer, *Busseola fusca* (Lepidoptera: Noctuidae) with special reference to insect-plant interactions. Insects.

[7] Tabashnik B.E. (1994). Evolution of resistance to *Bacillus thuringiensis*. Annu. Rev. Entomol..

[8] Gould F. (1998). Sustainability of transgenic insecticidal cultivars: Integrating pest genetics and ecology. Annu. Rev. Entomol..

[9] Gassmann A.J., Carrière Y., Tabashnik B.E. (2009). Fitness costs of insect resistance to *Bacillus thuringiensis*. Annu. Rev. Entomol..

[10] Carrière Y., Crowder D.W., Tabashnik B.E. (2010). Evolutionary ecology of insect adaptation to Bt crops. Evol. Appl..

[11] Siegfried B., Jurat-Fuentes J.L. (2016). Editorial overview: Pests and resistance: Resistance to Bt toxins in transgenic crops. Curr. Opin. Insect Sci..

[12] Bourguet D., Desquilbet M., Lemarié S. (2005). Regulating insect resistance management: The case of non-Bt corn refuges in the US. J. Environ. Manag..

[13] Tabashnik B.E., Carrière Y. (2017). Surge in insect resistance to transgenic crops and prospects for sustainability. Nat. Biotechnol..

[14] Van Rensburg J.B.J. (2007). First report of field resistance by the stem borer, *Busseola fusca* (Fuller) to Bt-transgenic maize. S. Afr. J. Plant Soil..

[15] Tabashnik B.E., Carrière Y. (2019). Global patterns of resistance to bt crops highlighting pink bollworm in the United States, China, and India. J. Econ. Entomol..

[16] Tabashnik B.E., Brevault T., Carrière Y. (2013). Insect resistance to Bt crops: Lessons from the first billion acres. Nat. Biotechnol..

[17] United States Environmental Protection Agency (USEPA) (1998). The Environmental Protection Agency’s White Paper on Bt Plant-Pesticide Resistance Management.

[18] Gould F. (2000). Testing Bt refuge strategies in the field. Nat. Biotechnol..

[19] Bates S.L., Zhao J.Z., Roush R.T., Shelton A.M. (2005). Insect resistance management in GM crops: Past, present and future. Nat. Biotechnol..

[20] Tabashnik B.E., Gassmann A., Crowder D.W., Carrière Y. (2008). Insect resistance to Bt crops: Evidence versus theory. Nat. Biotechnol..

[21] Tabashnik B.E., Van Rensburg J.B.J., Carrière Y. (2009). Field-evolved insect resistance to Bt crops: Definition, theory, and data. J. Econ. Entomol..

[22] Onstad D.W., Mitchell P.D., Hurley T.M., Lundgren J.G., Porter R.P., Krupke C.H., Spencer J.L., Difonzo C.D., Baute T.S., Hellmich R.L. (2011). Seeds of change: Corn seed mixtures for resistance management and integrated pest management. J. Econ. Entomol..

[23] Razze J.M., Mason C.E. (2012). Dispersal behavior of neonate European corn borer (Lepidoptera: Crambidae) on Bt corn. J. Econ. Entomol..

[24] Carroll M.W., Head G., Caprion M. (2012). When and where a seed mix refuge makes sense for managing insect resistance to Bt plants. J. Crop Prot..

[25] Carrière Y., Fabrick J.A., Tabashnik B.E. (2016). Can pyramids and seed mixtures delay resistance to Bt crops?. Trends Biotechnol..

[26] Van den Berg J. (2017). Wild host plants of stem borers cannot contribute to insect resistance management in Bt maize in Africa. J. Econ. Entomol..

[27] Van den Berg J., Hilbeck A., Bøhn T. (2013). Pest resistance to Cry1Ab Bt maize: Field resistance, contributing factors and lessons from South Africa. J. Crop Prot..

[28] Head G. Adapting insect resistance management strategies for transgenic Bt crops to developing world needs. Proceedings of the 8th International Symposium on the Biosafety of Genetically Modified Organisms (ISBGMO).

[29] Mulaa M.A., Bergvinson D., Mugo S., Ngeny J. (2007). Developing insect resistance management strategies for Bt maize in Kenya. Afr. Crop Sci. Conf. Proc..

[30] Tefera T., Mugo S., Mwimali M., Anani B., Tende R., Beyene Y., Gichuki S., Oikeh S.O., Nang’ayo F., Okeno J. (2016). Resistance of Bt-maize (MON810) against the stem borers *Busseola fusca* (Fuller) and *Chilo partellus* (Swinhoe) and its yield performance in Kenya. Crop Prot..

[31] Mallet J., Porter P. (1992). Preventing insect adaptation to insect-resistant crops: Are seed mixtures or refugia the best strategy?. Proc. R. Soc. Lond. Ser. B Biol. Sci..

[32] Pannuti L.E.R., Paula-Moraes S.V., Hunt T.E., Baldin E.L.L., Dana L., Malaquias J.V. (2016). Plant-to-plant movement of *Striacosta albicosta* (Lepidoptera: Noctuidae) and *Spodoptera frugiperda* (Lepidoptera: Noctuidae) in maize (*Zea mays*). J. Econ. Entomol..

[33] Onstad D.W., Crespo A.L.B., Pan Z., Crain P.R., Thompson S.D., Pilcher C.D., Sethi A. (2018). Blended refuge and insect resistance management for insecticidal corn. Environ. Entomol..

[34] Davis P.M., Onstad D.W. (2000). Seed mixtures as resistance management strategy for European corn borers (Lepidoptera: Crambidae) infesting transgenic corn expressing Cry1Ab protein. J. Econ. Entomol..

[35] Heuberger S., Crowder D.W., Brévault T., Tabashnik B.E., Carrière Y. (2011). Modeling the effects of plant-to-plant gene flow, larval behavior, and refuge size on pest resistance to Bt cotton. Environ. Entomol..

[36] Ives A.R., Glaum P.R., Ziebarth N.L., Andow D.A. (2011). The evolution of resistance to two-toxin pyramid transgenic crops. Ecol. Appl..

[37] Brévault T., Tabashnik B.E., Carrière Y. (2015). A seed mixture increases dominance of resistance to Bt cotton in *Helicoverpa zea*. Sci. Rep..

[38] Carrière Y., Dutilleul P., Ellers-Kirk C., Pedersen B., Haller S., Antilla L., Dennehy T.J., Tabashnik B.E. (2004). Sources, sinks and the zone of influence of refuges for managing insect resistance to Bt crops. Ecol. Appl..

[39] Bernays E.A., Chapman R.F. (1994). Host-Plant Selection by Phytophagous Insects.

[40] Sauvion N., Thiéry D., Calatayud P.A. (2017). Insect-Plant Interactions in a Crop Protection Perspective. Advances in Botanical Research.

[41] Visser A., Du Plessis H., Erasmus A., Van den Berg J. (2019). Preference of Bt-resistant and susceptible *Busseola fusca* moths and larvae for Bt and non-Bt maize. Entomol. Exp. Appl..

[42] Zalucki M.P., Clarke A.R., Malcolm S.B. (2002). Ecology and behaviour of first instar larval Lepidoptera. Annu. Rev. Entomol..

[43] Head G.P., Greenplate J. (2012). The design and implementation of insect resistance management programs for Bt crops. GM Crops Food.

[44] Thompson J.A. (2008). The role of biotechnology for agricultural sustainability in Africa. Philos. Trans. R. Soc. Lond. B. Biol. Sci..

[45] Aheto D.W., Bøhn T., Breckling B., Van den Berg J., Ching L.L., Wikmark O., Breckling B., Verhoeven R. (2013). Implications of GM crops in subsistence-based agricultural systems in Africa. GM Crop Cultivation-Ecological Effects in a Landscape Scale. Theorie in Der Ökologie, 17.

[46] Kotey D.A., Assefa Y., Van den Berg J. (2017). Enhancing smallholder farmers’ awareness of GM maize technology, management practices and compliance to stewardship requirements in the Eastern Cape Province of South Africa: The role of public extension and advisory services. S. Afr. J. Agric. Ext..

[47] MacIntosh S.C. (2009). Managing the risk of insect resistance to transgenic insect control traits: Practical approaches in local environments. Pest. Manag. Sci..

[48] Assefa Y., Van den Berg J. (2010). Genetically modified maize: Adoption practices of small-scale farmers in South Africa and implications for resource-poor farmers on the continent. Asp. Appl. Biol..

[49] Jacobson K., Myhr A.I. (2012). GM crops and smallholders: Biosafety and local practice. J. Environ. Dev..

[50] Strydom E., Erasmus A., Du Plessis H., Van den Berg J. (2018). Resistance status of *Busseola fusca* (Lepidoptera: Noctuidae) populations to single- and stacked-gene Bt maize in South Africa. J. Econ. Entomol..

[51] Kotey D.A., Obi A., Assefa Y., Erasmus A., Van den Berg J. (2017). Monitoring resistance to Bt maize in field populations of *Busseola fusca* (Fuller) (Lepidoptera: Noctuidae) from smallholder farms in the Eastern Cape Province of South Africa. Afr. Entomol..

[52] Van Rensburg J.B.J., Van Rensburg G.D.J. (1993). Laboratory production of *Busseola fusca* (Fuller) (Lepidoptera: Noctuidae) and techniques for the detection of resistance in maize plants. Afr. Entomol..

[53] Solomon M.E. (1951). Control of humidity with potassium hydroxide, sulphuric acid or other solutions. Bull. Entomol. Res..

[54] Goldstein J.A., Mason C.E., Pesek J. (2010). Dispersal and movement behavior of neonate European corn borer (Lepidoptera: Crambidae) on non-Bt and transgenic Bt corn. J. Econ. Entomol..

[55] Ramalho F.S., Pachú J.K.S., Lira A.C.S., Malaquias J.B., Zanuncio J.C., Fernandes F.S. (2014). Feeding and dispersal behavior of the cotton leafworm, *Alabama argillacea* (Hübner) (Lepidoptera: Noctuidae), on Bt and non-Bt cotton: Implications for evolution and resistance management. PLoS ONE.

[56] Calatayud P.A., Ahuya P.O., Groutte S., Le Rü B. (2015). The first hours in the life of a *Busseola fusca* (Lepidoptera: Noctuidae) larva. Entomol. Ornithol. Herpetol..

[57] Vélez A.M., Alves A.P., Blankenship E.E., Siegfried B.D. (2016). Effect of Cry1F maize on the behavior of susceptible and resistant *Spodoptera frugiperda* and *Ostrinia nubilalis*. Entomol. Exp. Appl..

[58] Han P., Velasco-Hernández M.C., Ramirez-Romero R., Desneux N. (2016). Behavioral effects of insect-resistant genetically modified crops on phytophagous and beneficial arthropods: A review. J. Pest. Sci..

[59] Swamy S.V.S.G., Sharma H.C., Subbaratman G.V., Vijay M.P. (2008). Ovipositional and feeding preferences of *Helicoverpa armigera* towards putative transgenic and non-transgenic pigeonpeas. Resist. Pest. Manag. Newsl..

[60] Schwartz J.M., Tabashnik B.E., Johnson M.W. (1991). Behavioral and physiological responses of susceptible and resistant diamondback moth larvae to Bacillus thuringiensis. Entomol. Exp. Appl..

[61] Hoy C.W., Hall F.R. (1993). Feeding behaviour of *Plutella xylostella* and *Leptinotarsa decemlineata* on leaves treated with *Bacillus thuringiensis* and esfenvalerate. Pestic. Sci..

[62] Ramachandran S., Buntin G.D., All J.N., Tabashnik B.E., Raymer P.L., Adang M.J., Pulliam D.A., Stewart C.N. (1998). Survival, development, and oviposition of resistant diamondback moth (Lepidoptera: Plutellidae) on transgenic canola producing a Bacillus thuringiensis toxin. J. Econ. Entomol..

[63] Ramachandran R., Raffa K.E., Miller M.J., Ellis D.D., McCown B.H. (1993). Behavioral responses and sublethal effects of spruce budworm (Lepidoptera: Tortricidae) and fall webworm (Lepidoptera: Arctiidae) larvae to *Bacillus thuringiensis* Cry1A(a) toxin diet. Environ. Entomol..

[64] Berdegué M., Trumble J.T., Moar W.J. (1996). Effect of CryIC toxin from *Bacillus thuringiensis* on larval feeding behaviour of *Spodoptera exigua*. Entomol. Exp. Appl..

[65] Assefa Y., Dlamini T. (2016). Determining genetic variations in B. fusca Fuller (Lepidoptera: Noctuidae) and *Chilo partellus* Swinhoe (Lepidoptera: Crambidae) from Swaziland and South Africa through sequences of the mtDNA cytochrome oxidase sub-unit 1 gene. Int. J. Adv. Res. Biol. Sci..

[66] Campagne P., Capdevielle-Dulac C., Pasquet R., Cornell S.J., Kruger M., Silvain J.F., Le Rü B., Van den Berg J. (2017). Genetic hitch-hiking and resistance evolution to transgenic Bt toxins: Insights from the African stalk borer Busseola fusca (Noctuidae). Heredity.

[67] Peterson B., Bezuidenhout C.C., Van Den Berg J. (2016). Cytochrome c oxidase I and cytochrome b gene sequences indicate low genetic diversity in South African Busseola fusca (Lepidoptera: Noctuidae) from maize. Afr. Entomol..

